# Management Practices Promoting Sustained Implementation of the Quality Register Senior Alert for Older Adults in Municipal Care in Sweden

**DOI:** 10.2174/1874434601812010215

**Published:** 2018-10-24

**Authors:** Summer Meranius, Josefsson Karin

**Affiliations:** 1 School of Health Care and Social Welfare, Mälardalen University, Västerås, Sweden; 2 Faculty of Caring Science, Work Life and Social Welfare, University of Borås, Borås, Sweden

**Keywords:** Elderly care, Leadership, Management, Quality register, Sustainable development, Health care

## Abstract

**Background:**

Senior Alert is a national quality register aimed at supporting a standardized, structured, and systematic preventive care process for adults aged 65 and over in the areas malnutrition, pressure ulcers, falls, problems with oral health and bladder dysfunction. Therefore, the quality register is particularly suitable for older adults with multimorbidity.

**Aim:**

The aim was to describe management practices that contributed to the sustained implementation of the quality register Senior Alert in municipal elderly care in Sweden.

**Methods:**

The design of this pilot study was descriptive and inductive. The sample of *n* = 12 included managers (*n* = 7) and care staff (*n* = 5) at seven municipal care homes for older adults in Sweden. The study was performed between April 2014 and June 2014 using two methods: Individual interviews and nonparticipant unstructured observations. Data were analyzed using qualitative content analysis.

**Results:**

The analysis led to the following generic categories: leading teamwork, leading a preventive care process and leading a supportive organizational structure, and to one main category: management promoting learning and quality improvement.

**Conclusion:**

To be sustainable, Senior Alert implementations in municipal elderly care need management. Management, by leading teamwork, a preventive care process and a supportive organizational structure, is essential for achieving learning and quality improvement.

## 1 INTRODUCTION

Sweden is divided into 290 municipalities and 20 county councils including hospitals and healthcare centers [1]. County councils and municipalities have their own self-governing local authorities responsible for different activities. Responsibility for the health, social care, home and special accommodation services for adults aged 65 and over rests with Sweden’s municipalities.

Sweden and Italy have Europe’s largest proportions of older adults. In 2015, 20% of the current Swedish population was 65 or older [2], *i.e.* over 1.9 million older adults, and of these almost half a million are older than 80. The expected increase in the number of older adults poses a healthcare challenge [3]. Forecasts indicate that the cost of care for older adults in Sweden may increase by 270% by 2040. One challenge is that older adults report more health problems compared with health professionals’ needs assessments [4]. Health, health promotion and equal treatment are key factors in promoting healthy aging.

In Sweden, national quality registers have recently been established in health and medical services to ensure better outcomes for patients, improve professional development and provide better functioning systems [5]. Quality registers are often disease-specific, for example, the hip and cataract. Senior Alert (SA) is one exception, a registry for preventive care process regarding falls, pressure ulcers, malnutrition, oral health and bladder dysfunction [6].

**Fig. (1):**
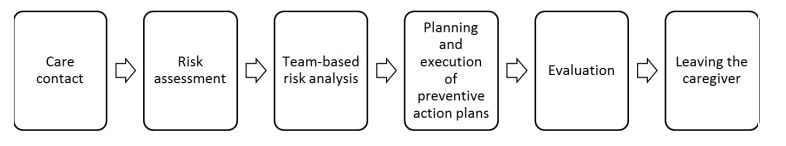
The six steps of the Senior Alert preventing care process.

Senior Alert (SA) is a national quality register aimed at supporting a standardized, structured, and systematic preventive care process for adults aged 65 and over but could be used for younger people. SA is accessible *via* the internet, and the preventive process contains six steps: 1) care contact; 2) risk assessment; 3) team-based analysis of risks; 4) planning and execution of preventive action plans; 5) evaluation; and 6) leaving the caregiver (Fig. **1**).

The healthcare professionals can register the patient and next of kin’ self-reported answers during their meeting or use a paper version of the instrument for later registration [7, 8]. Functional outcomes are important in a quality register [9] since simple self-reported items can be transformed by care staff into a modified ranking scale and used with high precision in the future for comparing care results when developing care delivery and health improvements. The SA quality registry has a focus on population health among older adults, but it also supports an individualized, standardized, and systematic preventive care process for this group [8].

Using SA, municipalities and county councils want to develop a new preventive approach that increases the potential for the best possible health and social care regardless of who provides it [7]. SA also provides the government and healthcare politicians with data for setting goals for elderly care [8]. SA is used by 90% of Swedish municipalities and county councils. The total number of risk assessments completed from 2009 to 2014 exceeded 1 000 000.

Decision makers need to organize care in a more sustainable way [10]. There is an urgent need of sustainability in municipal care since the numbers of older adults are expected to increase dependent of care, along with a shortage of care staff [10]. Sustainability is defined as the continuation or the integration of new practices in an organization. It has become a routine in care and also continues to be a support in delivering desired outcomes [11]. There are theoretical models of sustainability in practice change, such as the National Health Service Institute for Innovation and Improvement Sustainability Model (SM) [12]. The SM supports teams implementing new practices in their work by addressing sustainability. The SM aims to facilitate the ability of teams to identify and self-assess key factors in their local context that help define whether a new practice will be sustainable. Thus, the SM might increase the probability that sustainability can be achieved. There is increasing evidence for this in terms of leadership in implementation [13]. Leaders’ attitudes, priorities, and behavior [14] are increasingly being seen as major contributors to outcomes for employees and organizations [13, 15] suggested that leadership behaviors represent orientations such as being able to guide the implementation process properly, being proactive in responding to implementation needs, being supportive to care staff during the introduction of new practices, and persevering in addressing obstacles that are expected to stall implementation efforts.

Considering that the number of older adults is increasing and that older age correlates with health problems, systematic preventive care processes are needed, and functional outcomes in a quality register are important. Risk assessment as a basic in-care preventive process aims to strengthen health and well-being both in individuals and in populations of older adults. However, there is limited research describing factors needed for sustainable implementations of SA.

The aim was to describe management practices that contribute to the sustained implementation of SA in municipal elderly care.

## 2 METHODS

### 2.1 Design

A descriptive and inductive design was used [16]. The study was performed between 201404 and 201406 using a cross-sectional design comprising two methods: individual interviews and nonparticipant unstructured observations [16]. The sample of 12 included managers (*n* = 7) and care staff (*n* = 5) knowledgeable of and responsible for SA at seven municipal care homes for older adults in Sweden. Data were analyzed using a qualitative content analysis method [17]. The project was approved by a regional cooperation group with a municipality and a university in central Sweden.

### 2.2 Setting and Sample

Sweden has three political assemblies, municipal, county council and regional, and a national parliament [1]. Health and social care are politically steered by two main laws, the Health and Medical Services Act and the Social Services Act.

Sampling for selected cases that met a predetermined criterion of importance was applied [16]. The study’s settings were chosen with the help of SA’s registrar and head statistician who provided information on all municipal elderly homes (*n* = 35) in Sweden that have successfully implemented SA. A successful SA implementation entails: 1) SA being integrated throughout the care process, from assessment to evaluation of malnutrition, falls, pressure ulcers, impaired oral health and bladder dysfunction in older adults; 2) team-based assessments; 3) team-based investigations; and 4) the results of all measures taken for SA-assessed older adult patients are evaluated. To achieve a geographical distribution in this study, eight municipal elderly homes were initially included.

The managers of these homes were emailed a letter informing them that their SA was successful and that they had been chosen to join the study. They were asked if the researchers could call and explain more about the study. All managers were contacted again and provided with oral and written information about the study. All gave their informed consent to the study. One home was excluded since they were unable to attend. Managers selected team members who were knowledgeable of and responsible for SA at the elderly care homes as potential informants. The team members received written information from the researcher (MSM) about the study and gave their informed consent to participate.

The total numbers of participants were *n* = 12 (1 each from 4 sites, 2 each from 4 sites); managers (*n* = 7), registered nurses (*n* = 3), an enrolled nurse (*n* = 1), and a physiotherapist (*n* = 1). All were knowledgeable of, responsible for and had worked with SA. All participants were female with a median age of 50 (range, 48 - 65). Five had education in leadership. The managers had worked 10 years (median) in care units (range = 3 - 30 years). They had worked for three years (median) at their current workplace (range = 1 - 20 years). All participants had been leading the implementation of SA in their units based on their professional roles.

### 2.3 Data Collection

#### 2.3.1 Interviews

The participants (*n* = 12) were interviewed separately at their workplaces for 60–90 minutes. Seven interviews were face to face and five by telephone due to geographic distance [16]. The goal in conducting individual interviews was to encourage free expression about the issue. The participants were asked one open question, “Can you talk about how you worked with SA at your workplace?” Supplementary questions were used as needed, including: A) Can you give examples? B) What do you think about this? C) Can you elaborate on that and tell me more? All interviews were recorded and transcribed verbatim. The criterion sampling contributed to the immediate identification of relevant informants, and data saturation was achieved when no new information was obtained [16].

#### 2.3.2 Nonparticipant Unstructured Observations

Nonparticipant unstructured observations [16] were performed to supplement the interviews and validate what had been said. Observations were made at three team meetings at three municipal elderly homes. The meetings were audio-recorded and notes were taken. The team meetings were attended by all enrolled nurses, registered nurses, physiotherapists, occupational therapists and managers. After the meetings, supplementary questions were asked to clarify the understanding of terms used during the SA assessment. These three sites were selected based on willingness to participate, ability to hold a team meeting within a reasonable time frame, as well as geographical location. Team meetings lasted 1.45 to 2 hours. Managers gave information about the schedules of team meetings.

### 2.4 Data Analysis

Data were analyzed using qualitative content analysis [17]. The analysis processes are represented as three main phases: preparing, organizing and reporting. Interview analysis took an inductive approach; in other words, the categories were not predetermined; rather, they emerged through the analysis. The analysis was abstract, as in a process of description on an abstract level, going from open coding to creating subcategories, generic categories and the main category. Striving for objectivity is about oscillating between the parts and the whole, not digressing. Table **1** shows an example of the data analysis. After analysis, a categorization matrix was developed (Table **2**) and all data from the observations were reviewed and compared in a deductive way toward the subcategories in Table **2**. The subcategories that could not be validated by observations were **s**taff continuity and **c**lear targets as identified and articulated goals at different levels within the organization. No further categories were added.

### 2.5 Ethical Considerations

No ethical approval is required in Sweden for research on staff members. Permission for the study was obtained from a regional cooperation group with a municipality and a university in central Sweden. The participants gave their informed consent to participate, were informed that their participation was voluntary, that data collected would be handled confidentially, and that they were free to withdraw from the project at any time. Further ethical aspects, based on ethical guidelines for human and social research, were followed throughout the study [18].

## 3 RESULTS

The main results showed that sustainable implementations of SA in municipal elderly care require managers who lead: teamwork, a preventive care process and a supportive organizational structure. They also require management to promote learning and quality improvement.

All categories were permeated with common ethical values, responsibility, reflective communication and patient-centered care. The participants stated that common ethical values ​​gave a quality register an ethical dimension by maintaining focus on the older adults and their complex and individual needs. It also emerged that the team clarified responsibility and responsibility enabled teamwork. Everyone took responsibility based on their profession and they focused on the older adults, while this process required manager support. Patient-centered care was important when working with SA as SA is based on the needs of older adults, focused on their entire life situation. Reflective communication was also essential, as it created understanding for the quality register. Reflective communication created preconditions for a critical approach, daring to try new ways of working and making visible shortcomings, thus triggering learning processes. It also created preconditions for team confidence by detecting shortcomings in the quality register.

### 3.1 Leading Teamwork

#### 3.1.1 A Present and Supportive Manager

The results indicated the importance of managers supporting their teams both during the SA implementation and after in its continuous use and development. Managers created the team dynamic, especially for enrolled nurses who dared take their place in the team and feel confident. By attending team meetings, managers could gather information about the quality of care, as well as lead the care based on values.

*It is important that I participate in everything. Participating as a manager also shows that the work with the quality register is important …it is easy for the work to fall into oblivion if managers don’t participate…that I know.* (Manager)

Awakening curiosity and reflection, and problematizing nursing were considered as pursuing efforts to increase future quality. The goal of managers was for everyone to be able to take responsibility for their actions without any defensiveness.

*When enrolled nurses and nurse aids recount their experiences, I ask ‘how do you see this and how have you thought of that’ and so on. Sometimes I need to do that, interrupt a little. But I talk a lot about the importance of everyone participating.”* (Manager)

In the team, everyone worked together toward common goals. A present and supportive manager helped enable team members’ competencies in benefiting the older adults.

#### 3.1.2 Importance of Teamwork and Team Competence

Teamwork was a prerequisite for working with SA. Managers supported their teams' development from the team being created to it functioning well and continuing to function. Different competencies were needed for team success. It was, therefore, important that everyone, regardless of education, felt important in the team. Managers created dynamic teams of mainly enrolled nurses who dared take their place in the group and felt confident. Registered nurses supported enrolled nurses in their contact with older adults and this process was supported by managers. The following quotes are examples of the importance of teamwork.

*I always participate in team meetings. I believe that team meetings are the most important meetings for me as a manager…because they are rewarding. That’s what’s most important and I can drive direction and show value base according to my mission.* (Manager)

*We show each other how we have worked with the quality register, its results, so it really becomes part of the implementation plan which we’re working on ... so it doesn’t just fall into oblivion. We keep the register alive, remember and discuss it at all daily morning meetings.* (Enrolled nurse)

The teams promoted learning and team members strengthened their individual professional roles. When making a joint assessment, everyone's knowledge was used. It was important to lead the team’s attitude so that team members focused on the older adults’ needs. The team became a team when everyone was aware of and worked together toward common goals. Reflective communication was described as a way to develop team responsibility as reflection facilitated the disposition to dare to try new approaches and pinpoint shortcomings. It also promoted learning, which was stated as possibly leading to a more confident team, which, in turn, facilitated taking responsibility. This can be described as the team clarifying responsibilities and accountability and enabling teamwork with everyone taking responsibility based on their profession and focusing on the older adults. This process needed to be supported by the manager. Another key-factor in leading teams was described as common basic values and patient-centered health and social care. This motivated and imbued the team’s work and, at the same time, gave the SA work an ethical dimension, by focusing on older adults’ individual needs. The following quotes are examples of team competence.

*The team meetings we have are great; you can educate and inform, you can demonstrate different techniques and everything. Enrolled nurses especially acquire a way of thinking, it feels like ... let’s take rehabilitation, me and the occupational therapist and the nurse, we’ve got it. But enrolled nurses also start thinking about risk; they learn to see…before the event, for example, a wound.* (Physiotherapist)

*We thought it was so awesome to see and, as they said, ‘wow, what a trip we’ve been on.’ We tested different things, we were at loggerheads ... it was emotional. But we dared to test the ideas that came up and said that we’d run it for three months and evaluate if it works. Now, enrolled nurses are much more confident in their roles and that is also what the team is for.* (Manager)

### 3.2 Leading a Preventive Care Process

Leading a preventive care process was important when working with SA; having clear targets, understanding the current situation, measuring results and following-up. Working with SA, to achieve both patient-related and organization-wide quality, required joint and overarching practice-led and operational objectives that needed to be established and communicated. It was important to identify and articulate the goals on different levels to drive quality improvement work forward. It was also important to create both joint and overarching practice-led and operational objectives on different levels within the organization. These then needed to be established and communicated. Understanding the current situation was a prerequisite for implementing a preventive process, measuring results and following-up, as well as being deemed an important starting point for reflections on other success factors. Anchoring results and following-up were the responsibility of all staff and required continuous and sustained efforts. Understanding results and following-up could lead to learning and quality development for the organization. Working on results and following-up were difficult and required support from various levels within the organization, from managers to medically responsible nurses.

*We had many patients who fell. So, we formed a team, which included all the professional categories; the older adults had physio regularly and we noticed good results. We worked in teams, measuring their balance, re-evaluating and we got really good results. What we also noticed was the teamwork ... that enrolled nurses had to learn balance exercises. And they continued the practice daily with the older adults. It’s been over a month, and none of the older adults have fallen because they are more active.* (Physiotherapist)

*I have noticed that the word ‘why’, is a loaded word, usually in a negative way. But how else can we reflect on why we ought to work with the quality register? It often turn to defending the word ‘why’ which obstructs reflection. When I become defensive, I can never reflect ... so my strategy is to transform the word to a positive affirmative word.* (Manager)

The SA work needed to be secured, followed-up, and that was everyone’s responsibility. Reflective communication promoted the understanding of the SA results, which could lead to learning and quality development. To succeed with the preventive care process, managers described once again the importance of shared ethical values and a preventive care process based on the needs of the older adults, which focused on their entire life situation. Patient-centered and reflective communication promoted a critical approach which is essential for good care quality.

*I want everyone to know that this work is so important for our older adults, and for care quality. We are here for the patient and, therefore, we work with quality, and that can help us too.* (Manager)

*We have always focused on the needs of the older adults, but now we have more track of each person. The entire team is on the right track and understand. Probably not everyone used to work the same way, based on the older adults. Rather…I’d do it my way and, when she was working, she did her way. But since we’ve discussed the older adults, and everyone gets to know what they want, it’s become better for them. The team helps to accomplish this.* (Manager)

### 3.3 Leading a Supportive Organizational Structure

Leading a supportive organizational structure was important when working with SA. Key-factors were: staff continuity, staff education, establishing meeting times and technical equipment. To work with SA, staff needed the support of a quality developer of registers or equivalent. Creating a meeting structure in which quality registers were anchored in the daily, weekly and monthly meetings was essential. Schedules were established so that everyone could participate. All staff needed education and information about the rules when working with SA. The team understood the value of there being more than one person who could record work in SA.

It was also important that there was a supportive structure during meetings, to help stay on topic and within the specified time. The participants pointed out that staff had to take responsibility for using supportive structures, such as sticking to the meeting agenda, absorbing information, participating in training, and having the patience and interest needed to make SA work. Access to technical equipment like laptops and computer screens facilitated the SA work.

*We have a quality developer who has helped us throughout, but now I think we could do it ourselves. I mean all staff are well aware of what’s what. And I plan it in their work schedules; on this day, there’ll be a follow-up. Since we have developed templates for what contact persons should check... weight, and maybe blood pressure, and such.* (Manager)

*In fact, I think it requires a certain type of good working atmosphere to have the strength to pursue these kinds of issues regarding quality registers, because they take time and you have to ask for the results and whether the care staff are using the register, then, if they do the first assessment of talking with the patient, it takes dedication and drive to, and holding out.* (Registered nurse)

## 4 DISCUSSION

This pilot study describes how sustainable SA implementations in municipal elderly care involve management practices promoting learning and quality improvement. This study demonstrates the importance of managers leading teamwork, a preventive care process and a supportive organizational structure for the sustainable implementation of SA. According to the World Health Organization [19], many countries need to make radical changes to health promotion and care for older adults. In order to achieve this, municipalities are expected to develop effective nursing management. Thus, it is crucial to provide managers with distinct and supportive organizational prerequisites [20].

Leading teamwork was a key factor for sustainable SA implementations; this included a present and supporting manager. It seems important to organize elderly care in such a way that managers are able to be present in care staffs’ daily practice [21]. Support, knowledge, involvement and time are key issues that can help care staff to work more constructively using guidelines such as a quality register in their daily practice. Care staff needs supportive managers who can help them solve various work-related problems and prioritize the daily care of older adults. Managers need clear organizational prerequisites [20]. However, there have been examples of registered nurses in municipal elderly care perceiving organizational prerequisites as unclear. Thus, managers need a long-term strategy to enable them to become effective team leaders and implement quality improvement in a clinical setting.

The results show that a sustainable SA implementation involves teamwork and team competence. This can be described as the team clarifying responsibility and responsibility enabling teamwork, while everyone takes responsibility based on their profession and focuses on the older adults. Our findings are confirmed by earlier research [22] that team-based reflection can enable a change which affects the organization and helps professionals to make sustainable improvements. The importance of including all staff members in the process of implementing guidelines is consistent with earlier research [23]. That this process needs to be supported by managers has been confirmed by research [20, 24]. Previous research emphasized that staff in municipal elderly care perceived a highly strenuous work situation characterized by high demands, such as high time pressure [20, 21, 24]. Therefore is staff support of importance in municipal elderly care, since it has a buffering effect in relation to the occurrence of work-related stress, for example, that a manager shows care, pays attention and creates team spirit [20, 24]. Support also includes fellow team members expressing positive feedback, helping and showing personal interest.

This study shows that leading a preventive care process is an influential factor in sustainable SA implementations. Key-factors were having clear targets, understanding the current situation, measuring results and following-up. Leading the SA preventive care process starts by mapping risks, focusing on falls, pressure ulcers, malnutrition, oral health and bladder dysfunction [7, 8]; after which planned and implemented interventions need to be followed-up. Management is crucial for accomplishing the full preventive process, from implementation to continuous use and development [8]. Therefore, assisting clinical nursing leaders in managing all aspects of quality, structure, process and outcome is of value [25].

Leading a supportive organizational structure was an influential factor in sustainable SA implementations in municipal elderly care. The study’s results show that the participants defined a supportive organization as staff continuity, staff education, established meeting times and technical equipment.

Recent research has pointed out that care staff perceives guidelines, such as quality registers, as not sufficiently anchored in their workplaces [21]. They describe how they do not receive sufficient education on the guidelines and that education seems to be a key issue for successful implementation of guidelines. Care staff also lacks other resources, such as technical equipment, with not enough computers in order to work according to guidelines. We agree with Batalden, * et al*. [26], we must figure out how to best prepare tomorrow’s healthcare workforce. As things stand, there are frustrations with improvements that do not last. We believe leading a supportive organizational structure can create the conditions for teamwork and a preventive care process, which promote learning and quality development. Research also shows that staff in municipal elderly care should be empowered, acknowledged and supported [21, 24, 27]. Nevertheless, little is known about interventions aiming to develop managers’ leadership skills in facilitating implementation [28].

Management without appropriate leadership rarely functions well. Inappropriate leadership that does not effectively delegate and acknowledge down the line is just as troublesome.

## 5 LIMITATIONS

The choice of unstructured nonparticipant observations in this study was based on the assumption that observers can see group interactions and activities [16]. The limitation of such observations was obvious when the subcategories Staff continuity and Clear targets, intended to identify and articulate goals at different levels within the organization, were not validated in the team meetings, but arose in the interviews. Even though SA is often a responsibility of registered nurses, only one enrolled nurse participated in the interviews, which contributed to a limitation of the study. This was compensated for in the team meetings where enrolled nurses were in the majority because of the staffing situation in elderly care homes, and because the remaining subcategories from the interviews validated the team meetings.

The small sample of participants and the Swedish context is a threat to transferability. However, the participants all worked in different workplaces, which varied in size and were distributed throughout a region, so others working with SA may identify with the results. There is always a risk for informants to feel exposed when raising negative aspects and to feel they are obliged to participate in a study. Because the participants in this study were selected from successful elderly care homes, they took significant pride in being able to share their experiences with SA. There were no indications in this situation that they might have felt obliged to participate in the study; instead, they felt acknowledged for the hard work they had been doing. Negative aspects were raised during the data collection and might confirm that the informants felt free to express both positive and negative experiences. No ethical problems or conflicts occurred during the study.

## CONCLUSION

Implementations of SA in municipal elderly care need management to be sustainable; they should focus on leading: teamwork, a preventive care process and a supportive organizational structure.

## Figures and Tables

**Table 1:** Example of the qualitative content analysis process.

**Text Unit**	**Code**	**Sub-Category**	**Generic Category**	**Main Category**
The team is important and it is important that the team is led, otherwise it will fail. Different competencies come together. No matter what your role, you are important, and it is important to emphasize that you are very important regardless of your education.	Teamwork is important for success of Senior Alert (quality register)	Importance of teamwork	Leading teamwork	Management to promote learning and quality improvement
	Everybody’s competencies are needed in the team regardless of profession	Team competence		

**Table 2:** Overview **of the results via** sub-categories, generic categories and main categories.

**Sub-Category**	**Generic Category**	**Main Category**
Present Manager	Leading teamwork	Management to promote learning and quality improvement
Supportive Manager		
Importance of Teamwork		
Team Competence		
Clear Targets	Leading a Preventive Care Process	
Understanding the Current Situation		
Results and Follow-up		
Staff Continuity	Leading a supportive organizational structure	
Staff Education		
Established Meeting times		
Technical Equipment		

## CLINICAL IMPLICATIONS

This study identifies factors that influence sustainable implementations of quality registers. It is essential that managers recognize their decisive leading roles in sustainable SA implementations in municipal elderly care. Managers should promote learning and quality improvement and should especially focus on leading: teamwork, a preventive care process and a supportive organizational structure.
